# Ectopic ACTH Secretion in a Child With Metastatic Ewing's Sarcoma: A Case Report

**DOI:** 10.3389/fonc.2020.00574

**Published:** 2020-04-28

**Authors:** Valentina Di Ruscio, Giada Del Baldo, Maria Debora De Pasquale, Rita De Vito, Evelina Miele, Giovanna Stefania Colafati, Annalisa Deodati, Maria Antonietta De Ioris, Assunta Tornesello, Giuseppe Maria Milano, Angela Mastronuzzi

**Affiliations:** ^1^Department of Paediatric Haematology/Oncology, IRCCS Bambino Gesù Children's Hospital, Rome, Italy; ^2^Department of Paediatric, Sapienza University of Rome, Rome, Italy; ^3^Department of Pathology, Bambino Gesù Children Hospital (IRCCS), Rome, Italy; ^4^Department of Paediatrics, Bambino Gesù Children Hospital (IRCSS), Palidoro, Italy; ^5^Oncology Hospital Vito Fazzi, Lecce, Italy

**Keywords:** Ewing's sarcoma, paraneoplastic syndrome, Cushing Syndrome, ACTH, pediatric oncology

## Abstract

Ectopic ACTH syndrome is rare in pediatric age. Sarcomas that cause Ectopic ACTH Syndrome (EAS) are even more uncommon. Currently, only three cases of EAS caused by Ewing' sarcoma have been reported. We detail a 10-year-old boy with Cushing's syndrome symptoms caused by ectopic ACTH production by a metastatic Ewing's sarcoma of the right ischio-pubic and ileo-pubic branches. The rapid appearance of cushingoid symptoms, with significant weight gain, acne, hirsutism, and hypercortisolism were implications of ectopic ACTH production as paraneoplastic Cushing's Syndrome. The very high levels of ACTH and non-suppression at the high dose dexamethasone test confirmed the clinical suspicion. We underline the possibility EAS was caused by an ACTH-secreting tumor, including soft tissue sarcomas.

## Introduction

Paraneoplastic Cushing's syndrome (CS) is an unusual cause of hypercortisolism due to ectopic ACTH secretion by non-pituitary mass (1). Ectopic ACTH-producing tumors rarely occur in children, with <1% of all adolescents with CS (2). Many cases of ACTH-secreting ectopic neuroendocrine tumors are reported in literature (3, 4). In most patients, the tumors secrete corticotropin-like peptides and/or corticotropin releasing factor (CRF)-like peptide, which stimulate cortisol hyperproduction (4).

In adults, the most common tumors causing ectopic ACTH Syndrome (EAS) are bronchial neuroendocrine tumors, carcinoid of thymus, pancreatic carcinoma and neural tumors (5). In pediatric age, the tumors that most frequently cause an EAS are neuroblastomas and neuroendocrine neoplasms, while in adolescents, are carcinoid tumors, both sporadic and in the context of multiple endocrine malignancies (6). Soft tissue sarcomas causing EAS are uncommon (7, 8). We report a case of a child with EAS due to metastatic Ewing's sarcoma.

## Case Report

A 10-year-old boy presented to our institution with a history of rapid weight gain (8 kilos in the past 4 months), recent onset widespread acne, hirsutism, depression, and muscle weakness. His height was −0.87 SDS, weight +0.54 SDS and body mass index (BMI) SDS was +1.21. During the physical examination we observed facial plethora and fullness, lower limb edema, dorso-cervical fat pad (“buffalo hump”), hirsutism, obesity and hypertension (BP 135/80 mmHg, > 99° pct).

Blood tests showed fasting hyperglycemia (180 mg/dl) and high levels of ACTH and plasma cortisol according to circadian rhythm (at 00.00 am: ACTH 34,1 pg/ml, range 0–42, cortisol 63,84 mcg/ml, range 4–22, at 8.00 a.m.: ACTH: 62,7 pg/ml—cortisol: 84,96 mcg/dl). We performed a high-dose suppression test with 8 mg of dexamethasone, without suppression of adrenal axis. A significant glycosuria was revealed by a single urine spot. High levels of free urinary cortisol was detected in a 24-h urine collection (>510 mcg/24 h).

Ultrasonography and abdominal magnetic resonance imaging (MRI) showed bilateral surrenalic hyperplasia with pseudonodular appearance of the upper portion of left adrenal gland.

**Figure 1:**
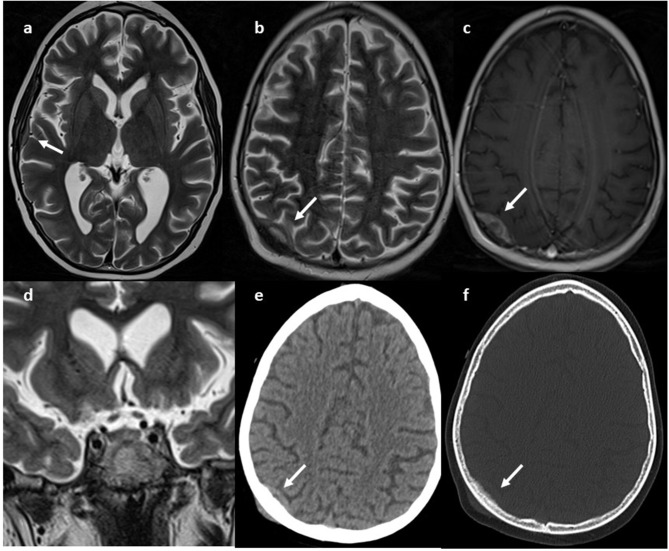
MRI. Axial **(a,b)** and coronal **(d)** T2w and axial GdT1 **(c)** images show two skull lesions located in the right parietal and temporal region, with intense contrast-enhancement. Pituitary gland was normal (**e**, white arrow) CT axial images **(e,f)** show infiltrative aspect of the lesion.

During the first few days after admission, the patient developed several epileptic fits with hypotony, paresthesia of the left upper arm and generalized hypertonia, that required antiepileptic therapy. Brain MRI showed a small lesion in the right occipital region, without significant mass effect, and another lesion in the right temporal region; the pituitary gland appeared normal (Figure 1).

Within a few days, an intense lombar pain appeared. Due to the suspicion of a osteoporotic vertebral fracture, we decided to perform a total spine radiography which identified a lesion of the right ischio-pubic and ileo-pubic branches.

**Figure 2:**
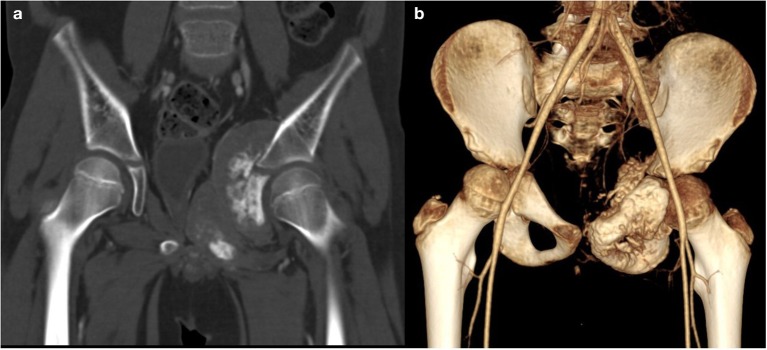
CT. Coronal (**a**, bone window) and 3D **(b)** reconstruction show a voluminous tumor involving the left ischio-and ileo-public branches with extensive bone destruction, speculated periosteal reaction, and partially mineralized soft tissue mass.

A total body computerized tomography (CT) scan showed an infiltrative localization of the right ischio-pubic and ileo-pubic branches. A bone scintigraphy confirmed multiple secondary bone localizations (vault and skull, skull-base, spine, several ribs, sternum, left scapula, pelvis, both femurs, and the right tibia) (Figure 2).

**Figure 3:**
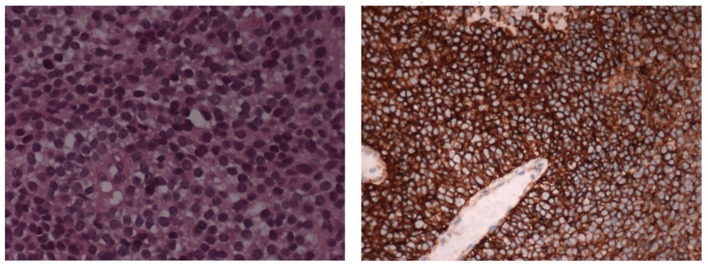
The histological picture is a monomorphic proliferation of small round cells with scanty cytoplasm containing glycogen (E&E 10X). The cells shows positive stain for CD99 (immunoperoxidase stain 10X).

A biopsy of the mass confirmed Ewing's Sarcoma with EWS/FLI-1 gene fusion (9) diagnosis (Figure 3). The bone marrow biopsy showed a secondary localization of tumor.

The patient was subsequently enrolled on the ongoing national protocol of the Associazione Italiana di Ematologia ed Oncologia Pediatrica (AIEOP) ISG EW2 for metastatic Ewing's sarcoma (10).

After the first course of chemotherapy, the ACTH and cortisol blood levels normalized (cortisol 4,65 microgr/ml, ACTH 37,3 pg/ml), and the clinical signs of hypercortisolism almost completely disappeared.

In the subsequent 2 months, the neurological symptoms became worse, with daily epileptic seizures, severe hypotonia and left hemiparesis. The brain CT scan showed a progressive disease correlated by abnormal ACTH levels (50.9 pg/ml; Figure 3).

We decided to switch to a second line therapy, with two courses of high-doses ifosfamide.

Unfortunately, 4 months later, the patient died for progressive metastatic disease.

## Discussion

Cushing's syndrome is uncommon, especially in children, with an overall prevalence of 2–5 new cases per million people per year, 10% concerning pediatric age (2, 11).

Symptoms of Cushing's disease in children may be different from that observed in adults. Usually, patients show significant signs of hyper-cortisolism and protein hyper-catabolism, such as obesity, hypertension, osteoporosis with fractures, hypertrichosis, infections, peripheral neuropathy and psychiatric disorders (12). Generally, the metabolic alterations are more evident, such as hypokalemia, hyperglycemia and metabolic alkalosis.

CS is classified into ACTH-independent and ACTH-dependent causes. The ACTH-indipendent CS is mainly related to iatrogenic causes, excessive administration of exogenous cortisol, or adrenal glands alterations (hyperplasia, adenoma, or carcinoma). ACTH-dependent CS may be caused by a pituitary adenoma secreting ACTH (a specific condition known as Cushing's disease) (13), that is the most common endogenous cause, but also by a surrenalic adenoma or carcinoma, or an ectopic ACTH production (EAS).

The term EAS was proposed by Liddle in 1963; it was based mainly on observation of patients with small cell lung cancer (SCLC). Since then, scientific research describes paraneoplastic CS in association especially with bronchial neuroendocrine tumors, thymic neuroendocrine neoplasms, thymoma but also Wilms tumor (14, 15), clear cell renal sarcoma, pheochromocytoma (16), neuroblastoma (17, 18), oncocytic renal carcinoid (19), clear cell sarcoma and carcinoid tumor arising in the duodenum (20, 21). In ~10–20% of cases, we are unable to discover the underlying primary tumor, and the cause of EAS remains unknown (22).

Scientific writings express EAS as the main cause in a few percentage of pediatric CS (7), ~10%, (23, 24) and it is even rarer among paraneoplastic syndromes (PS) (Table 1).

**Table 1:** Causes of EAS.

**Common**	**Rare**	**Very rare**
Bronchial neuroendocrine neoplasms Thymoma, thymic neuroendocrine neoplasms Lung cancer Pancreatic/gastrointestinal tract neuroendocrine tumors Medullary thyroid cancer Pheochromocytoma	Ovarian cancer Colorectal cancer Prostate cancer Cervical cancer Neuroblastoma	Esophageal cancer Kidney cancer Hepatocarcinoma Breast cancer Salivary gland tumor Pleural mesothelioma Soft tissue tumors Lymphomas Malignant melanoma Ovarian and testicular Laryngeal tumor

In children with EAS, the most frequent tumors are bronchial or thymic, but also oncocytic renal carcinoid (17) and a carcinoid tumor arising in the duodenum, Wilms tumor (14, 15), adrenal neuroblastoma (18), clear cell sarcoma have been reported.

PS usually develops in a restricted percentage of patients with tumors (5). There are various mechanisms underlying PS. Frequently, tumors induce auto-immunity or immune complex production; in a few cases, they may produce biologically active peptides such as growth factors, enzymes, unidentified humoral factors or, as in our patient, hormones such as ACTH (14).

A diagnostic workup for the screening of Cushing' syndromes (paraneoplastic or not) consists of several steps. The Endocrine Society guidelines recommend after a proper clinical examination and routine laboratory tests, a 24 h free urinary cortisol dosing, a late night salivary cortisol test and a dexamethasone suppression test (DST) as the first examinations (20).

Since arriving at our institution, the patient showed several symptoms and signs of Cushing's syndrome, such as proximal muscle weakness, modest changes in fat distribution, with dorso-cervical fat pad, systolic hypertension, rapid weight gain and widespread hirsutism (7). The laboratory findings showed hypernatremia and hypokalemia, indicating mineralocorticoid effects of extreme hypercortisolism. Blood cortisol was elevated, such as the free urinary level; the circadian rhythm was interrupted, suggesting the diagnosis of CS. The dexamethasone suppression test did not reduce the blood cortisol level and the 24-h urinary 17-hydroxycorticosteroid, confirming the diagnosis of EAS (7). In fact, a high dose of dexamethasone usually exerts negative feedback on pituitary neoplastic ACTH-producing cells, but not on ectopic ACTH-producing cells or adrenal adenoma (12). As the second step, a CRH stimulation can be performed and it may be helpful in order to distinguish between a pituitary or ectopic cause, because CRH receptors are only expressed on the pituitary gland (25).

The imaging evaluation should include not only a brain MRI to detect pituitary tumors and a PET/TC using 18F-FDG or 68 Ga-labeled somatostatins analogs (26), but also total body CT scans to allow a complete patient' staging.

We decided to perform first an ultrasonography and abdominal magnetic resonance imaging (MRI) on our patient, showing bilateral surrenalic hyperplasia with pseudonodular appearance of the upper portion of left adrenal gland. During the first days of admission, a brain MRI was carried out, due to the sudden appearance of neurological symptoms, with several epileptic fits, hypotony, paresthesia of the left upper arm and generalized hypertonia. The MRI showed two small lesions in right occipital and temporal region and the subsequent total body CT scan identified a mass infiltrating the right ischio-pubic and ileo-pubic branches. A fine needle biopsy of the mass established the diagnosis of Ewing' sarcoma. These results supported the empirical hypothesis of a paraneoplastic Cushing' syndrome caused by EAS of Ewing's tumor cells.

For what concerned therapy, in literature, aside from surgical and medical treatment of the underlying disease, there are many first-line pharmacologic options for paraneoplastic CS. The large part of these drugs inhibits steroid production. These drugs include ketoconazole, mitotane, metyrapone, and aminoglutethimide (27). Despite several collateral effects, such as nausea, hepatotoxicity, and hypogonadism, ketoconazole is usually the most tolerated (22); instead, metyrapone, which results in eucortisolemia in 50% of patients, may cause hirsutism. Mifepristone, which competes with the glucocorticoid receptor, has recently shown excellent results in both clinical and laboratory data (27). Mitotane, with its cytotoxic effect on adrenocortical cells, can be used for as long term therapy and in double or triple combinations with other drugs (25). Anti-hypertensive agents and diuretics are other therapeutic options (27, 28).

Other treatments include somatostatin analogs (octreotide, lantreotide e pasireotide). They have an antiproliferative and an antisecretory effect, but with a high risk of resistance on long term therapy. In case of severe hypercortisolemia, a surgical approach is suggested when medical treatments fail (22).

Our patient did not received a pharmacological therapy for hypercortisolism and its symptoms, except of an anti-hypertensive agent for systolic hypertension. After the start of the chemotherapy, we detected a significant recovery of the circadian cortisol rhythm, a reduction of circulating ACTH levels and free cortisol in urine spot, and a rapid normalization of blood pressure values; thus strongly consolidated that the source of the EAS was the tumor.

Respect to classic CS, in EAS, the duration of the symptoms is <6 months (7). As in our patient, it is usually associated with a worse prognosis, due to the aggressiveness of the underlying disease.

Nowadays, only three cases of EAS syndrome caused by an Ewing' sarcoma among pediatric patients are reported in literature.

The first case report described a 12-year-old girl with CS caused by Ewing's sarcoma of the tibia secreting CRH-like peptide. Symptoms and biochemical findings of Cushing's syndrome regressed after surgery (7)_._

The second case reported was a 9-year-old girl with a history of rapid weight gain, acne and acanthosis of neck skin (8). Laboratory findings confirmed the suspicion of CS. Abdominal ultrasound and abdomen MRI revealed a large retroperitoneal mass. The biopsy confirmed the Ewing sarcoma diagnosis. The patient received combination chemotherapy with vincristine, adriamycine, cyclophosphamide and radiotherapy (8). After ten courses of chemotherapy in 6 months, abdominopelvic MRI revealed a reduction in the tumor and a surgical performed. Cushing's syndrome disappeared after 6 months of treatment (8).

The third case reported a 9-year-old boy with a long history of an increasing mass on the left distal thigh, weight gain, acne and acanthosis nigricans (29). He showed the typical clinical signs of CS. Blood pressure levels was high. At laboratory tests, they evaluated electrolytes alterations, metabolic alkalosis and high levels of serum cortisol and ACTH. Low and high dose dexamethasone suppression tests failed (29). MRI of hypothalamus-pituitary region was normal. The biopsy of the thigh mass was consistent with Ewing Sarcoma. The patient received standard chemotherapy with Vincristine, Doxorubicin, and Cyclophosphamide (29).

The final outcomes of the first two patients are unknown, whereas the third patient died of sepsis 5 days after start of chemotherapy.

In our report, the fourth known case to date, we detailed the history of a 10-year-old boy with several signs and symptoms of Cushing's syndrome. A high dose of dexamethasone failed to suppress the high cortisol levels, thus suggesting an ectopic origin of hypercortisolism. We performed a brain MRI for the sudden appearance of neurological symptoms, which showed two small lesions in right occipital and temporal region. The total body CT scan identified a mass infiltrating the right ischio-pubic and ileo-pubic branches. A fine needle biopsy of the mass finally established the diagnosis of Ewing' sarcoma. The patient received standard chemotherapy according to the ongoing National Protocol. In the subsequent 2 months, after an initial clinical and laboratory response, the neurological manifestations became worse. The brain CT scan showed a significant increase in the size of the cerebral lesions. Four months later, he died of progressive metastatic disease, after two cycles of second-line therapy.

## Concluding Remarks

PS are rare conditions among pediatric patients. Soft tissue tumors causing EAS are even more rare. The rapid appearance of hypercortisolism symptoms with clinical and laboratory findings that showed high blood and urinary cortisol levels, high ACTH levels and a negative dexamethasone suppression test, are alerts to the possibility of EAS by an ACTH-secreting tumors, including the most rare, specifically, as in this case, soft tissue sarcomas. Imaging evaluations should always include total body scans, which allow the clinicians to detect even smaller lesions, far away from the adrenal gland. A biopsy is absolutely required for diagnosis. In most cases, front line chemotherapy normalize cortisol levels and mitigate clinical symptoms of hypercortisolism. Regardless, the prognosis remains poor due to aggressiveness of the underlying disease.

## Ethics Statement

Written informed consent was obtained from the minor(s)' legal guardian/next of kin for the publication of any potentially identifiable images or data included in this article.

## Author Contributions

AM has the original idea to report the case and treatment purposes and she coordinated the working group. GM, MDD, EM, and AD managed the patient. RD defined hystological details and molecular characterization. GC performed image acquisitions and review. VD and GD wrote the paper. MAD provided the English revision. All authors revised and approved final version of the manuscript.

## Conflict of Interest

The authors declare that the research was conducted in the absence of any commercial or financial relationships that could be construed as a potential conflict of interest.
